# Involving Families in Cardiac Care Through Remote Patient and Family Management: Focus Group and Journey Mapping Study

**DOI:** 10.2196/83055

**Published:** 2026-07-28

**Authors:** Julian Houwen, Veronica R Janssen, Sara M Hondmann, Niels H Chavannes, Maaike Kleinsmann, Douwe E Atsma, Valeria Pannunzio

**Affiliations:** 1 Department of Cardiology, Leiden University Medical Center, Albinusdreef 2, Leiden, 2333 ZA, The Netherlands, 31 643991522; 2 Department of Design, Organization and Strategy, Faculty of Industrial Design Engineering, Delft University of Technology, Delft, The Netherlands; 3 Unit of Health, Medical and Neuropsychology, Faculty of Social and Behavioral Sciences, Leiden University, Leiden, The Netherlands; 4 Department of Public Health and Primary Care (PHEG), Leiden University Medical Center, Leiden, The Netherlands; 5 National eHealth Living Lab, Leiden University Medical Center, Leiden, The Netherlands

**Keywords:** family-centered care, cardiovascular disease, remote patient management, journey mapping, digital health, unmet needs

## Abstract

**Background:**

In cardiovascular care, illness and recovery affect both patients and their families, particularly within home-based remote patient management (RPM). A recent scientific statement from the American Heart Association highlighted the importance of family involvement, identifying digital technologies as a key enabling opportunity. Despite this, research into the needs of families and the implications of RPM remains limited.

**Objective:**

This study explored the lived experiences and unmet needs of patients with cardiovascular disease (CVD) and their relatives within RPM-supported cardiac care, using perioperative care and myocardial infarction as representative trajectories. Based on the identified gaps, we proposed a set of features for remote patient and family management (RPFM) interventions to address these needs.

**Methods:**

This qualitative study was conducted at a Dutch university hospital with over a decade of experience in RPM across CVD pathways. A human-centered design approach was employed, including focus groups with 24 participants (13 patients with CVD and 11 relatives). Data analysis followed a framework analysis approach, combining inductive theme identification with deductive mapping onto existing frameworks. Care experiences and unmet needs were identified inductively and segmented along the Family Systems Illness Model’s phases of illness. The needs were subsequently categorized deductively into the domains of the Supportive Care Framework. Based on these user-informed insights, RPFM features were generated through author ideation and internal team consensus. Finally, these experiences, needs, and features were synthesized into a visual journey map illustrating key care moments across 3 phases: preadmission, admission, and postadmission.

**Results:**

We identified 47 unmet needs across 6 domains: informational (n=13), psychoemotional (n=13), social (n=7), physical (n=7), practical (n=6), and spiritual (n=1). The most significant gaps, described as “black holes” in care and support, emerged during the preoperative waiting period and early home recovery, which were characterized by a lack of information and psychoemotional support. To address the identified unmet needs, we generated 33 RPFM intervention feature ideas. These RPFM features were mapped across care phases and grouped into 7 categories: dynamic pathway navigation (n=8), on-demand support and information (n=5), family well-being modules (n=5), medical translation and consultation support (n=4), safety and assurance monitoring (n=4), collaborative lifestyle management (n=4), and peer support platform (n=3).

**Conclusions:**

This study identified experience gaps in RPM-supported cardiac care. It showed that most unmet needs for care and support extend beyond hospital admission and discharge and that most health behaviors and recovery occur in the home context, within the patient’s relational ecosystem with loved ones. The proposed RPFM features provide preliminary directions for exploration toward a transition from individually focused monitoring to inclusive, family-centered care and management.

## Introduction

Digital health technologies, including remote patient management (RPM), are increasingly embedded in cardiovascular care pathways with the goal of improving clinical outcomes, facilitating patient self-management, and decreasing pressure on the health care system [1-4]. RPM enables continuous guidance, real-time monitoring, and early clinical interventions while encouraging active patient engagement in self-care and recovery [5-8]. In turn, active patient engagement enables a range of opportunities for sustained lifestyle change, which significantly reduces cardiovascular risk [9].

Despite these developments and the growing emphasis on home-based self-care, most cardiovascular care models remain focused on in-hospital, staff-delivered care, underemphasizing the broader psychosocial and relational contexts in which cardiovascular illness is experienced [10,11]. As a result, the role of informal caregivers, such as family, is often overlooked [12]. However, these individuals play a crucial role in terms of cardiovascular risk management, home-based care, medication adherence, symptom monitoring, and emotional support [13]. Yet, they are often unrecognized in care delivery systems and therefore underprepared for their responsibilities, despite evidence of their impact on care quality and patient outcomes [10,12,13].

In a recent scientific statement, the American Heart Association has pointed out the importance of involving family systems in cardiovascular care [14]. Here the term “family” is defined in a broad sense, including not only biological relatives but also close friends, neighbors, or others whom the patient wishes to involve. The statement identifies digital technologies, including telehealth, as a key opportunity to support this integration [14]. This direction aligns with the goals of European health systems, where aging populations and health care staff shortages are increasing dependence on informal care [15,16].

Thus, there is now a clear need to advance RPM into remote patient and family management (RPFM), a model that actively includes and supports families throughout the care trajectory. As such, there is an urgent need to understand how RPM-supported models of cardiovascular care can effectively involve relatives throughout the care pathway, empower them in their role, and support them as individuals coping with the cardiac illness of a loved one.

Yet, empirical research on the needs of relatives, the implications of RPM for their caregiving experience, and what is required to design family-centered digital interventions that respond to their real needs remains limited. This study addresses this gap by exploring the lived experiences of patients and their relatives following RPM-supported cardiac care provided by a major university hospital in the Netherlands and by translating these experiences into RPFM intervention features.

In this study, we build on earlier research on family involvement in cardiac RPM [17] and employ a human-centered design approach that involves patients, family members, clinical experts, and design experts in a meaningful and iterative manner throughout the research process [18]. Using focus groups and journey mapping [19-24], we identify user-informed key care moments, unmet needs, and opportunities for family-centered RPM. Our analytical framework draws on the Family Systems Illness Model [25] and the Supportive Care Framework [26] to situate informal caregiving within a broader relational and psychosocial context.

By studying these often-overlooked family experiences and designing meaningful solutions, we aim to contribute to the development of more inclusive, “real-life” RPM-enabled cardiovascular care pathways that support both patients and their relatives in effectively coping with the disease trajectory and lifestyle modification. In doing so, we aim to contribute to improving both experiential and health outcomes for patients and their loved ones.

## Methods

### Setting and Design

This qualitative study was conducted at a university hospital in the Netherlands, with over a decade of experience in RPM [8,27-31]. It explored the experiences of patients and their relatives in 2 distinct patient groups, selected as representative examples of cardiovascular disease (CVD) care pathways: acute myocardial infarction (MI) and elective cardiac surgery within a perioperative (peri-OP) care pathway. These care pathways, despite differences in clinical presentation, were selected to provide broad, varied insight into the experiences of the largest cardiology patient populations and their relatives. In addition, both are supported by the same RPM program, *The Box* [8,27-31], which enables hospital service-level comparison. *The Box* offers hybrid care using eHealth devices (eg, smartwatch, blood pressure monitor, digital scale, and thermometer) and a connected patient-facing mobile app. The service is currently used for remote home monitoring of patients’ vital parameters, enabling clinical oversight and timely intervention while reducing the need for in-person hospital visits. Now that the “monitoring” foundation is in place in hospital practice, a shift in the intervention toward “self-management” has been initiated. This study contributes to this iteration by offering recommendations that broaden the service functionalities through involving and integrating patients’ family environments.

To develop recommendations for RPFM interventions, a human-centered design approach was employed. The process involved identifying patient and family experiences through focus groups, from which user-informed unmet needs were extracted. These needs then informed the generation of RPFM intervention features through author ideation and internal team consensus. Finally, the results were synthesized into a visual journey map. As a last step, the journey map and features were shared with participants via email for validation; however, no feedback was received. The study followed the COREQ (Consolidated Criteria for Reporting Qualitative Research) guidelines for qualitative research reporting [32].

### Participants and Recruitment

Patients in the MI and peri-OP pathways who were ≥2 months after hospital admission and still in hospital-based follow-up through the hybrid care program were purposively sampled. We aimed for a sample size sufficient to conduct at least 6 focus groups because this amount was found to uncover 90% of relevant themes in previous research [33]. Participants were invited via email, followed by telephone confirmation. Participants were encouraged to bring a family member, caregiver, neighbor or friend closely involved in their care. Recruitment aimed for variation in age, gender, and caregiving relationships. There were no preexisting relationships between the researchers collecting the data and the participants.

### Focus Groups

Seven focus group sessions were conducted at the hospital in June 2024, divided by care pathway. In 6 sessions, patients and relatives participated in separate groups to enable open discussion. One session combined both groups for practical reasons, allowing complementary patient-relative perspectives. Group sizes ranged from 3 to 4 participants per session (see Multimedia Appendix 1 for an overview). No significant differences in participants’ candor or the depth of shared experiences were observed between the combined and separate formats.

Each focus group session lasted approximately 2 hours. Every session ended with an interview wrap-up to determine whether, according to the participants, the most important topics and points were covered. Moderation was carried out by 2 researchers (JH, a male PhD researcher and strategic designer, and SMH, a female PhD candidate and medical psychologist), supported by a female medical psychology intern.

Semistructured discussions were held around the “Care and Well-Being Journey” template (available in Multimedia Appendix 2). This is a participatory mapping tool designed for this study based on existing journey mapping approaches [19]. Discussions covered 3 phases: preadmission, admission, and postadmission. Participants discussed key moments, challenges, and support experiences across the need domains of the Supportive Care Framework [26]. Post-it notes were used to annotate experiences on the template.

### Data Analysis

The discussions were recorded, and the audio was transcribed. The transcripts were analyzed using a framework analysis approach [34], combining inductive theme identification with deductive mapping onto existing frameworks and adapted for the purpose of journey mapping. The analysis aimed to generate themes related to participants’ care experiences and unmet needs, which were later mapped onto a patient journey format [19,20]. To structure the analysis, we used 2 theoretical models: the Family Systems Illness Model’s phases of illness [25], which informed segmentation of family experiences across the care pathway, and the Supportive Care Framework [26], which provided domains for categorizing unmet needs.

The analysis was performed in 3 steps. First, after familiarization with the transcripts, coding was conducted inductively by 2 researchers (JH and VP, a female assistant professor). They assigned meaningful labels to transcript text segments related to care experiences and unmet needs while simultaneously tagging each code with its phase along the cardiac care pathway. This step was conducted in ATLAS.ti 2025 (ATLAS.ti GmbH) software. Second, the codes were clustered and refined across multiple rounds of discussion, moving from initial codes to broader themes. These themes were visually organized into a journey map structure using Miro (RealtimeBoard, Inc) software. Third, within each theme, specific unmet needs for patients, relatives, and families were deductively categorized into the domains of the Supportive Care Framework [26]. To better reflect the data, we made a minor adaptation to Fitch [26] domains: psychological and emotional needs were combined into a single psychoemotional category, as participants did not seem to distinguish between these aspects during discussion.

No formal data saturation assessment was performed. Instead, the process continued through multiple rounds of consensus building until the research team agreed that the themes accurately reflected the participants’ experiences. Any discrepancies were resolved through discussion. Throughout the analysis, the researchers engaged in ongoing reflexivity, using transdisciplinary team discussions to examine how their backgrounds in medicine and design influenced theme development and to mitigate individual professional biases.

### RPFM Features Ideation

The themes and corresponding unmet needs were used by the first author (JH) to ideate RPFM intervention features. These were iteratively refined through internal multidisciplinary team consensus discussions involving clinical experts (VRJ, a female associate professor and medical psychologist, and DEA, a male professor of cardiology) and design experts (VP and MK, a female professor of design for digital transformation). This design process ensured that the features were grounded in the lived experiences of families and aligned with clinical and psychosocial realities.

### Journey Mapping

Finally, the collected needs and corresponding RPFM feature ideas were mapped visually, with the map iterated through multiple rounds to improve consistency and readability. This journey map offers both an overview of unmet needs across patients’ and families’ current journeys and a summary of additional RPFM features that could improve specific touchpoints.

### Ethical Considerations

Ethical approval was granted by the institutional nWMO-div1 review board at the Leiden University Medical Center (DAT/tak/1002024). Written informed consent was obtained from all participants prior to participation. All collected data were deidentified. As a gesture of appreciation for their participation, each participant received a €15 (approximately US $16) gift card.

## Results

### Participant Characteristics

A total of 42 individuals were invited to participate in the study, comprising 22 patients with CVD and 20 relatives. Of these, 9 pairs of patients and relatives did not participate, citing reasons such as illness, forgetting the scheduled session, or providing no explanation. This resulted in the inclusion of 24 participants (13 patients with CVD and 11 relatives). All participants received care and support primarily from the same university medical center and affiliated rehabilitation center. An overview of participants’ characteristics is presented in Table 1.

**Table 1.** Demographics and clinical characteristics of patients with CVD^a^ and relatives (N=24) in the qualitative focus groups.

Characteristic	Patients with CVD (n=13)	Relatives (n=11)	Total (N=24)
Sex, n (%)			
Female	8 (62)	5 (45)	13 (54)
Male	5 (38)	6 (55)	11 (46)
CVD care path, n (%)			
Peri-OP^b^	6 (46)	5 (45)	11 (46)
MI^c^	7 (54)	6 (55)	13 (54)
Relation to patient, n			
Partner	—^d^	7	7
Child	—	2	2
Parent	—	2	2
Age group (years), mean (SD; range)^e^	60.8 (12.6; 30‐80)	57.3 (17.7; 20‐85)	59.2 (14.9; 20‐85)

a CVD: cardiovascular disease.

b Peri-OP: perioperative.

c MI: myocardial infarction.

d Not applicable.

e Mean and SD were estimated using the midpoints of the reported age ranges.

### Journey Map

Through our human-centered design approach, we mapped the real-life experiences of individuals and their families dealing with cardiac events and symptoms, cardiovascular procedures, and their aftermath. Based on the data from the focus group sessions, we distilled a set of unmet needs across these phases, which we then used to generate RPFM interventions through author ideation and internal team consensus. The results of both steps are presented in a comprehensive journey map that displays categorized needs and related RPFM ideas.

**Figure 1.**
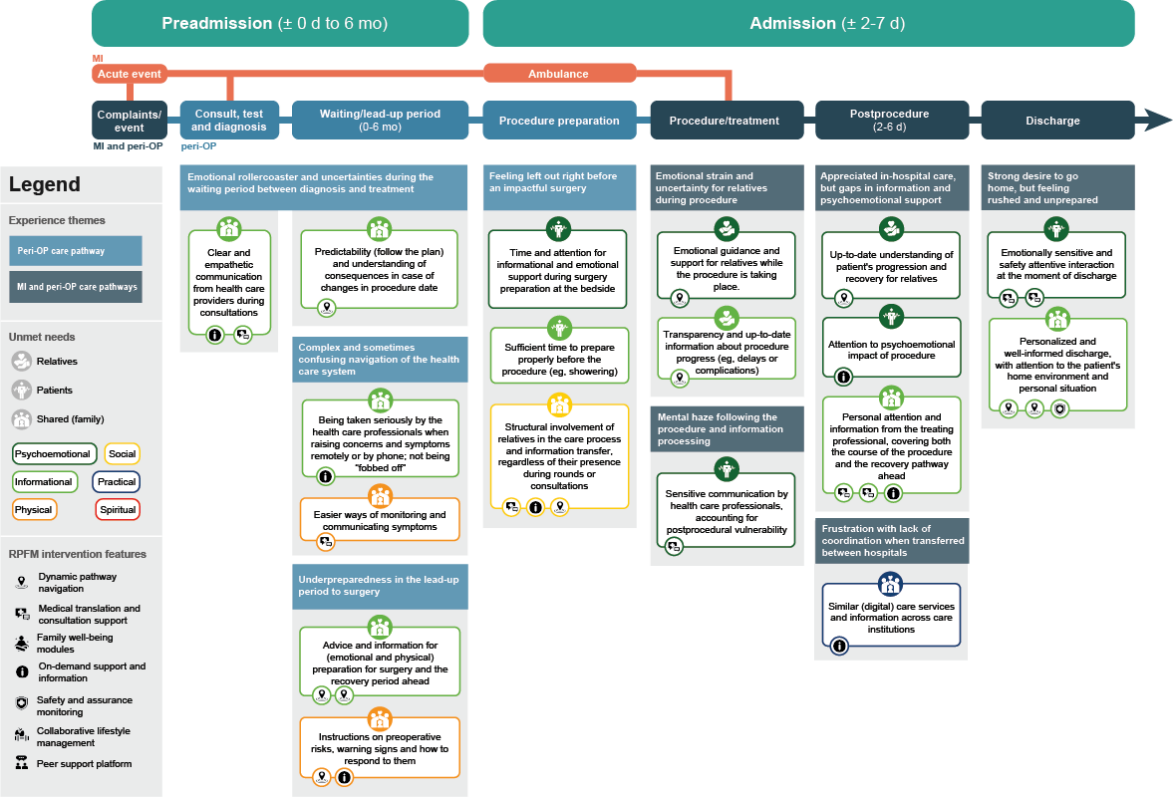
Journey map of the remote patient management–supported cardiovascular disease care pathway part 1: key phases, patient and relative experiences, unmet needs, and proposed RPFM intervention features. MI: myocardial infarction; peri-OP: perioperative; RPFM: remote patient and family management.

**Figure 2.**
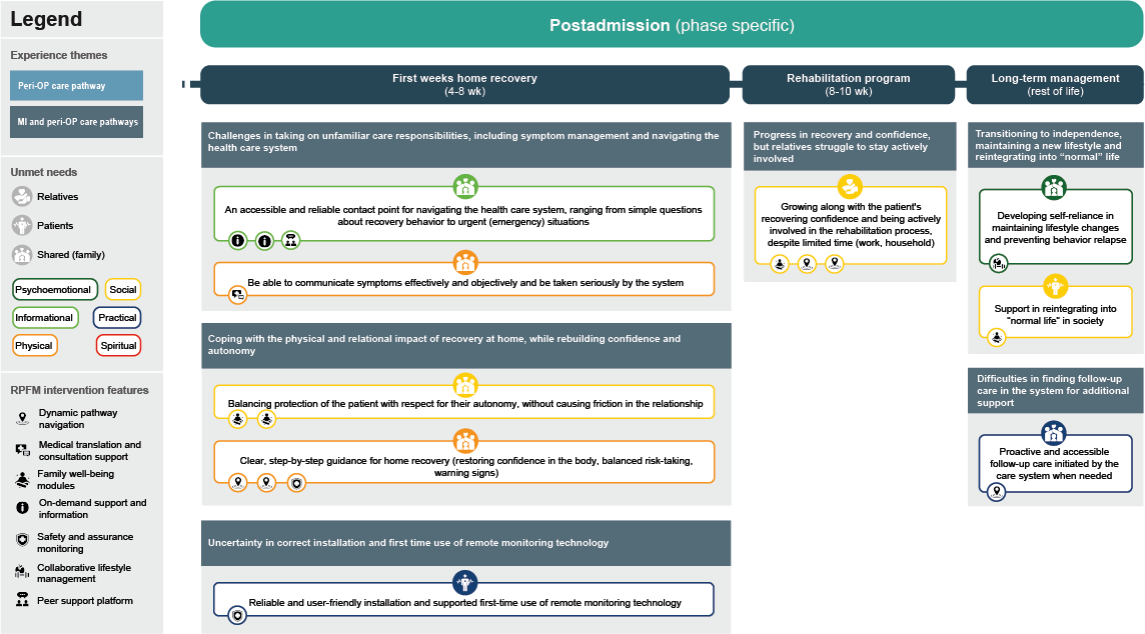
Journey map of the remote patient management–supported cardiovascular disease care pathway part 2: key phases, patient and relative experiences, unmet needs, and proposed RPFM intervention features. MI: myocardial infarction; peri-OP: perioperative; RPFM: remote patient and family management.

**Figure 3.**
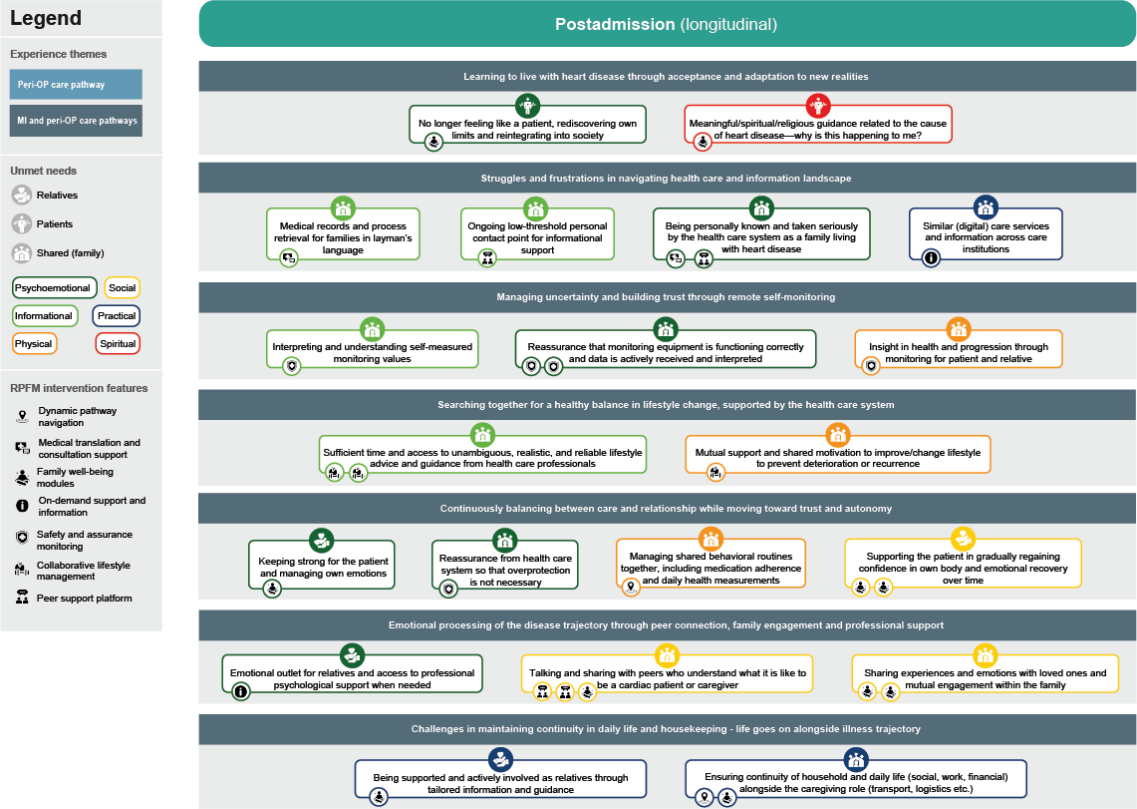
Journey map of the remote patient management–supported cardiovascular disease care pathway part 3: key phases, patient and relative experiences, unmet needs, and proposed RPFM intervention features. MI: myocardial infarction; peri-OP: perioperative; RPFM: remote patient and family management.

The full journey map is shown separately in Figures 1-3, visualizing a unified care pathway for MI and peri-OP care. Figure 1 illustrates the preadmission phase, including symptoms, diagnosis, waiting time, and the admission phase, spanning preprocedure to postprocedure treatment and discharge. Figure 2 presents postadmission with phase-specifics covering recovery at home, rehabilitation, and long-term management. Figure 3 also addresses the postadmission period, focusing on longitudinal experiences that could not be situated within a defined timeframe. In addition, within and across these phases, we identified unmet needs and presented them by experience themes, specifying whether they apply to patients, relatives, or families as a whole. These are color-coded by need domain (adapted from Fitch [26] Supportive Care Framework; see *Methods*). Additionally, RPFM intervention features to address these unmet needs are mapped in the journey (indicated by icons) under the related unmet needs.

### Unmet Needs and RPFM Feature Ideas

#### Overview

Our analysis revealed a broad range of unmet needs among patients, relatives, and families across different phases of the care pathways. A total of 47 unmet needs were identified and displayed in the journey map. These reflect significant gaps extending well beyond clinical management to the informational (n=13), psychoemotional (n=13), social (n=7), physical (n=7), practical (n=6), and spiritual (n=1) domains. Regarding the experienced impact of unmet needs, the most important phases were the “waiting/lead-up period” in preadmission (specifically peri-OP care) and the “first weeks of home rehabilitation” postadmission. These periods were considered by several participants to be “black holes” in care and support. During the admission, the unmet needs were experienced as less urgent, and clinical care was particularly praised.

Furthermore, the generation of human-centered ideas for RPFM features yielded 33 features applicable across the journey. These features were grouped into 7 main categories (see Table 2). A more detailed overview is available in Multimedia Appendix 3.

Some interventions are repeated throughout the journey as they respond to needs across multiple phases. These features are intended to enhance family-centered care by actively empowering family participation, improving information transmission, assisting navigation within health systems, aligning expectations, and providing holistic support throughout the cardiac care journey for patients and relatives.

Below is a detailed presentation of the key unmet needs, categorized by domain, along with examples of corresponding ideated RPFM features that may offer solutions.

**Table 2.** Ideated remote patient and family management (RPFM) intervention categories for patients with cardiovascular disease and relatives: descriptions, core focus, and RPFM intervention features.

Number	RPFM intervention categories	Core focus	Ideated RPFM intervention features
1	Dynamic pathway navigation (n=8)	What to do? What can we expect? How to do it?	Personalized recovery trajectory and goal planner Real-time guided expectation management Shared medical routine and event planner Joint health overview and progression Relatives’ rehabilitation insight feed Hospitalization support guidance Risk and symptom management instructions Follow-up care navigator
2	On-demand support and information (n=5)	Help us (now), please?	Emotional support and crisis access Digital emergency assistant Integrated family care-portal Intelligent information-assistance Knowledge navigator
3	Family well-being modules (n=5)	How do we cope and deal?	Social and practical support guide Caregiving guidebook Self-assessment check-ins Family dialogue toolkit Psychosocial resilience modules
4	Medical translation and consultation support (n=4)	Do we (families and the care system) understand each other?	Symptom and feeling translator Question logbook Family attendance scheduler Plain-language consultation archive
5	Safety and assurance monitoring (n=4)	Is it going well? Is it safe?	Discharge checklist Installation and connection validation Data-driven personalized feedback Automated monitoring feedback loop
6	Collaborative lifestyle management (n=4)	What can we do to prevent problems, and how do we get better?	Lifestyle information database Balanced and personalized lifestyle coaching Interpersonal motivational messaging Lifestyle assessment and feedback
7	Peer support platform (n=3)	Who do really understand us?	Buddy matching service Online peer support forum Virtual group sessions

#### Informational

Perioperatively, families reported inadequate communication from health care providers about the care process. They experienced uncertainty due to insufficient information (eg, about changes in procedure schedules and their impact) and felt that they were not taken seriously and were dismissed without clear explanations when expressing their concerns and questions. Guidance on behavioral, emotional, and physical preparation for the medical procedure and recovery was perceived as inadequate, with some families feeling rushed and unprepared due to limited consultation time and unclear instructions on expected behaviors and protocols. Family members also missed timely, transparent updates on the procedure’s progress, especially in cases of delays or complications.

In the preoperative phase, RPFM could support families in preparing for the unknown and reducing anxiety by providing clear information about the diagnosis and the entire care pathway. This could include summarized reviews of consultations and guided navigation to trusted sources. It may also offer preparation guidance and expectation management materials, such as videos explaining what to expect and advice on mutual physical and psychological readiness for the procedure, including lifestyle considerations. A personal care pathway tracker could offer families an overview of the care process. To further empower families to navigate the health care system, care navigation support could be included, for example, in the form of a decision tree indicating where and when to ask specific questions. On the day of the procedure, RPFM might deliver real-time digital updates in the event of delays or complications; for instance, if the procedure takes more time, a digital notification could be sent to the waiting family.

Postoperatively, patients missed direct and timely contact with their surgeon to discuss the process and outcome of the procedure. Families required more personalized information about the expected impact of heart disease on their care pathway and lives. Discharge from the hospital to home was often perceived as rushed, and the patient’s environment and circumstances were not sufficiently considered. At home, there was no easily accessible contact person for questions and support, nor was there clear guidance on navigating the health care system, especially in the case of alarming symptoms. Families also experienced problems accessing medical records from different organizations and needed explanations to understand them. Time to discuss lifestyle changes for recovery was often perceived as limited, with insufficient personalized advice and guidance.

In this postoperative phase, RPFM could support families with expectation management on both physical and mental recovery, alongside a discharge checklist and advice tailored to the home situation. Instead of receiving a confusing stack of papers, families could be provided with a personalized digital recovery plan outlining clear next steps and offering access to a digital Q&A system for immediate concerns. The platform could also offer a summary of the procedure’s course to clarify the patient’s “lost hours,” which is the period during which the patient was under anesthesia, and provide a step-by-step guide to the recovery pathway. For ongoing support, RPFM might feature a digital emergency assistant and facilitate access to low-threshold advice for informal guidance through a “buddy” system. It could also support personalized and balanced lifestyle coaching, facilitated by assessment tools and a trustworthy information database. Finally, RPFM could provide explanations of the collected data and offer a retrievable medical dossier summary in accessible language, empowering families to better understand the patient’s health status.

#### Psychoemotional

Families felt inadequately prepared for the care process due to limited consultation time and a lack of guidance regarding the emotional impact of the procedure. Both patients and their relatives experienced increased anxiety, stress, and frustration due to unclear or insufficiently empathetic communication by health care providers, especially during key moments such as consultation, hospitalization, discharge, and early recovery. There was also a sense of uncertainty in the reliability of the home monitoring systems due to limited feedback and doubt about whether data were being actively monitored by professionals.

Family members indicated that involvement in updates on the patient’s condition was limited, indirect, and not tailored to their emotional needs. During the recovery phase, both patients and family members struggled with the emotional impact of the illness and the effort to regain a sense of normality. Patients struggled to cope with “being a patient,” and with regaining emotional stability and autonomy. Family members felt inadequately prepared to support the coping process and reported a lack of accessible emotional support or structured channels to express their own fears, doubts, and emotional burden.

Patients and family members did not distinguish between psychological and emotional care. However, the analysis revealed a spectrum of illness-related distress. This ranged from general situational worries to potential clinical “red flags,” such as prolonged depressive thoughts and structural fears of recurrence when being alone. Discussing these needs even motivated a participant to express the need for additional mental health care from the hospital following the focus group session.

We propose using an RPFM approach to address these unmet psychoemotional needs, depending on severity. An example of more general support is providing digital updates to family members in an empathetic manner (content, tone of voice, and timing) that considers the stressful situation and emotional state, as well as providing guidance during impactful moments such as surgery. To build trust and reduce distress related to monitoring, RPFM could offer automatic reassurance notifications and proactive feedback, supported by personal data analysis. For more intensive support needs, it could provide direct emotional support avenues (eg, a crisis helpline), integrate individual emotional processing tools for relatives, and feature a mental health check. For broader coping and support, RPFM could facilitate peer connection through modules on living with chronic conditions, buddy systems, and shared experiences.

#### Social

Families reported insufficient opportunities for active involvement of relatives throughout the care journey and during information transfer. Relatives lacked support in balancing psychosocial care with patient autonomy and struggled to stay engaged in the care process due to their own responsibilities and other constraints. Relatives felt unequipped to assist patients and themselves in regaining trust in the body and in physical and emotional recovery and wished to be more involved in care. Support to adopt a healthier lifestyle together was often lacking, as was guidance on how to help the patient without causing friction. Also, the opportunity for family members to connect with peers for emotional or experience exchange was not accessible or structurally arranged.

To support greater social involvement and role clarity, RPFM could provide realistic expectation management for patients and relatives regarding active participation during admission. To navigate the delicate balance between support and patient autonomy, it could offer communication advice (eg, “How do I talk about this?”) and modules on discovering new boundaries for both patients and relatives (eg, a “Care & Release” coaching module that could support caregivers in transitioning from a period of intensive caregiving back to a more supportive role, allowing the patient to regain autonomy). RPFM could also include patient support training and parallel rehabilitation modules specifically for relatives, equipping them to provide effective, shared support. For sustained engagement and community building, RPFM could integrate an online peer support or matching platform for group or individual discussions for both patients and relatives, along with guidance on how to foster mutual support within the family unit.

#### Physical

Patients and families lacked concrete, step-by-step guidance for preparing for the medical procedure and home recovery, including support in restoring confidence in the body, safely weighing risk-taking, and recognizing and responding to warning signs. There was a need for simple, user-friendly tools to enable understanding, tracking, and communication of symptoms to health care providers. Furthermore, families required a shared understanding of recovery progress, medication adherence, and the patient’s emotional state to support understanding and confidence in both the body and the care process.

To meet these physical needs, RPFM could provide concrete guidelines for families to monitor and manage peri-OP and postoperative risks and symptoms, coupled with easy access to reliable information and a symptom “explainer” (eg, a pain/emotion score) for clear communication with the health care system. The platform could integrate rehabilitation modules and a personalized recovery planner, leveraging data to ensure that patients and families have a clear, shared understanding of the recovery journey. Furthermore, it could increase engagement through motivation modules and support for setting realistic, joint lifestyle change goals while also offering a shared health overview providing insight into progress and routines on medication or behavior change adherence.

#### Practical

Patients and relatives experienced practical issues due to insufficient data interoperability between institutions, which led to duplication and a lack of uniformity in care processes. Digital care services, such as the patient portal and installation, were frequently experienced as complicated or inconsistent, obstructing effective use. Relatives lacked remote access to touchpoints and information when they could not attend consultations due to work or household commitments. The provision of accessible, proactive follow-up care was perceived as inadequate. Additionally, there was limited practical support for accessing social services, for example, for maintaining daily household routines and managing finances alongside the ongoing care.

To address these practical challenges, RPFM could streamline care by providing a unified, user-friendly digital platform. It could offer a family-centered hub that ensures consistent information and centralized data storage and exchange. For instance, if a relative misses a consultation due to work, RPFM could offer remote access to summaries and a logbook for questions, empowering them to stay involved. It could also enable proactive care planning, with features such as a shared routine planner. Additionally, it could integrate digital guidance for social services and navigation of follow-up care in the region, easing the burden of managing daily life alongside care.

#### Spiritual

Finally, patients mentioned the lack of existential, religious, or spiritual guidance in processing and coping with the impact of the condition, a dimension that received little attention in the care pathway.

RPFM could support this through dedicated modules designed to offer guidance on dealing with existential questions and finding meaning.

## Discussion

### Principal Findings

Our study provides insights into the lived experiences of patients and their relatives following MI or peri-OP RPM-supported care pathways in a university hospital in the Netherlands. Through our human-centered design approach, we identified unmet psychoemotional, informational, social, practical, physical, and spiritual needs for both patients and their relatives. These results align with previous studies on family involvement in cardiovascular care [35-39] and deepen previous knowledge by detailing lived experiences across a broad spectrum of needs. Particularly, our results reinforce the importance of both improved information provision and improved psychological and emotional support to patients and family members. To achieve both goals, we recommend several RPFM intervention feature ideas for the current RPM-supported care pathways. In doing so, we directly address a research gap identified by Goldfarb et al [14], who highlight the potential of health technology to advance family engagement in cardiovascular care. It is important to note that the generated feature ideas, in their current state, are hypothetical, unvalidated prototypes rather than a finalized set of solutions ready for implementation.

Our results indicate that 2 stages in the pathway were particularly undersupported: the period prior to admission (for the peri-OP pathway) and the early recovery phase at home (for both pathways). These phases were described by patients and family members as “black holes” in care and were specifically characterized by a lack of information and psychoemotional support. These findings are consistent with previous research reporting similar challenges during the peri-OP and early recovery phases [40-43]. Medical professionals often focus on optimizing in-person encounters, such as consultations and admissions. However, we identified that, from a family perspective, the largest gaps in support and care occur during the extended periods spent at home. The missed support during these phases imposes a considerable burden on both patients and their relatives. Families sometimes feel abandoned and alone when it comes to managing fluctuating symptoms, taking over care responsibilities, and navigating the health care system. These challenges are intensified by the emotional impact of coping with heart disease while attempting to reestablish normalcy in daily life.

### Comparison to Prior Work and Implications

Illness and care cannot be separated from everyday life, in which patients live together with their loved ones in a caring ecosystem [25]. Most care, health behavior, and recovery take place outside professional settings [10,44]. It is, therefore, essential that the health care system extends its reach and actively supports these everyday contexts. This is especially important as hybrid home-based care models shift more care responsibility to patients and their family members. In that light, our results align with and expand upon the findings of Schmid et al [45]. They observed that peri-OP education is foundational in shaping cardiac patients’ subsequent behaviors and expectations regarding their treatment, particularly with respect to physical activity, nutrition, and mental health. Furthermore, they found that active patient and family engagement in the treatment process, including physiotherapy and mental support, significantly improves postoperative care and leads to faster recovery.

The proposed RPFM approach may represent a promising direction for addressing the identified gaps in care experiences, though it requires empirical validation. It offers hypothetical opportunities for continuous support and tailored interventions to be delivered directly into living environments. In this way, RPFM can reach patients and relatives at the moments when the “black holes” in information and support currently occur. Notably, these “black hole” phases overlap with the evidence-based window of opportunity where patients are most receptive to behavior change, as described in the framework of teachable moments [46]. Strategically supporting and educating families with RPFM interventions during these highly receptive periods could serve as a dual prevention mechanism, providing secondary prevention for patients and primary prevention for family members. This is particularly relevant as relatives may have similar risk profiles due to a common lifestyle, genetic predisposition, or shared environmental factors.

It is important to note that the identified unmet needs are not just “soft,” medically irrelevant experiential issues but have direct clinical and health system implications [47,48]. In this regard, we build on previous RPM research that describes the interconnectedness of patients’ experiences with other outcome measures [49]. By addressing the unmet needs in care and support reported by our participants, we hypothesize that the proposed RPFM approach could lead to outcome improvements across the components of the Quadruple Aim: health outcomes, patient experience, staff experience, and cost-effectiveness [50].

For instance, regarding health outcomes and cost-effectiveness, empowering families to recognize symptoms could improve clinical safety. For example, better-informed families might help identify early warning signs, which could theoretically reduce postoperative adverse events or prevent unplanned procedural postponements. Furthermore, RPFM’s ability to support joint remote management and lifestyle changes may also extend its benefits beyond the patient, promoting primary prevention for family members who may have similar risk profiles as the patients.

As for patient and family experience, RPFM could possibly directly address identified informational and psychological gaps. Tailored digital information on preoperative, postdischarge care, and recovery trajectories may enhance knowledge and navigation of care. To meet psychological needs, integrating therapeutic modules could address and support mental health. Through such features, RPFM could hypothetically help to mitigate anxiety, posttraumatic stress disorder, and depression, issues that, at times, affect both cardiac patients and their relatives [51,52]. These interventions may also reduce anxiety-driven overprotectiveness, which can hinder rehabilitation [53]. However, empirical testing is needed to establish whether such features would achieve these outcomes.

Finally, improving patient and family experience could directly impact staff experience. By reducing miscommunication and streamlining inquiries and support processes, RPFM could decrease staff workload, limiting repetitive inquiries and administrative friction.

### Future Directions

Overall, we propose shifting from home-based cardiac RPM to an RPFM approach that builds on the existing RPM infrastructure while also expanding it to explicitly integrate the family. This may include educational content, emotional support tools, and role-specific guidance tailored to the experiences of family members. This new paradigm acknowledges the relational dynamics of illness and recovery in home-based care models. Effectively involving families and meeting their needs can improve psychological health, self-efficacy, caregiving, quality of life, social support, and problem-coping skills. This, in turn, may lead to improved self-care and reduced hospital readmissions and unnecessary clinical visits [54]. However, empirical research is required to establish the impact and cost-effectiveness of the proposed RPFM interventions.

From a systems perspective, it is noteworthy that the 2 identified “black holes” in care and support needs (the preoperative waiting period and early home recovery) appear to align with the existing divisions between specialized hospital care and general practitioner care in the Dutch care system. At these transition points, families must navigate between different care environments while the breadth of support needs remains (eg, managing symptoms, seeking guidance, and balancing emotional and practical concerns). This occurs across care systems with different structures, protocols, and points of contact, contributing to the uncertainty already evident in these phases.

Here, an opportunity exists for RPFM to act as a connecting layer between the hospital and primary care by increasing the mobilization of informal family systems, whose involvement is constant throughout the different phases of the care journey. However, creating a coherent RPFM care pathway that is clear to patients and families, with clear guidance on where to seek care and support at each step, will require cross-boundary collaboration and integration between hospital and primary care providers. Future research should therefore actively develop and test RPFM-supported care pathways that operate seamlessly across care lines and engage multiple types of health care professionals. Furthermore, it is important to investigate the enabling conditions for this, for example, insurer reimbursement and data interoperability.

**Figure 4.**
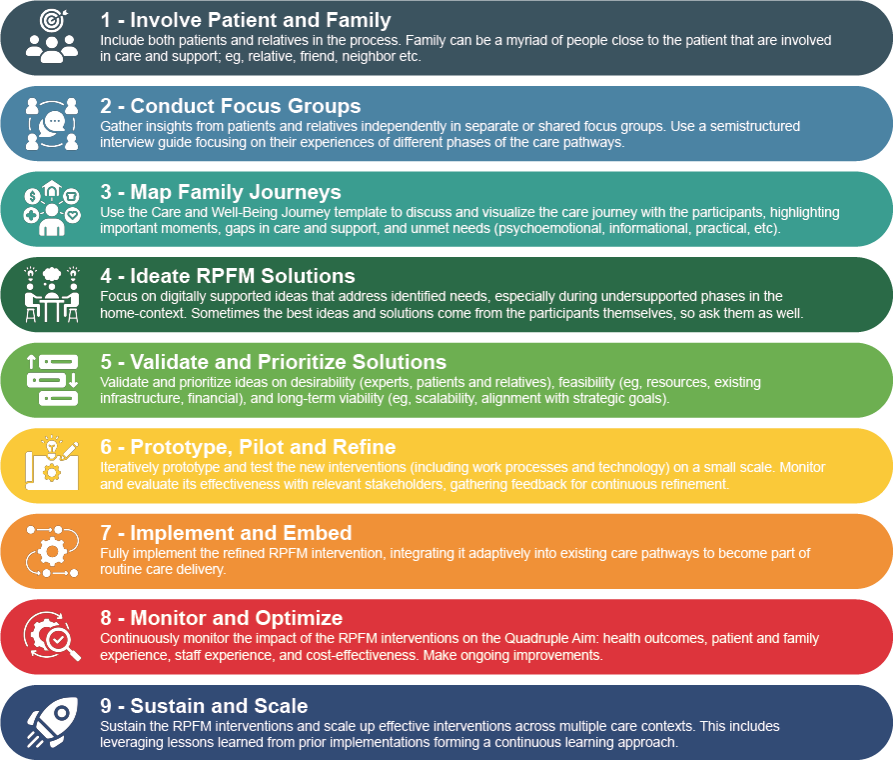
A proposed 9-step future innovation strategy for designing remote patient and family management interventions. RPFM: remote patient and family management.

Finally, reflecting on our human-centered design approach and identified opportunities, we propose an overall strategy for health care innovators wanting to design RPFM interventions. The strategy is presented in Figure 4. It offers a set of actionable steps, starting with an in-depth, empathetic understanding of patients and families through focus group discussions and journey mapping and concluding with the scaling of effective interventions across other care pathways. This overview also illustrates our plans for future RPFM research in relation to The Box [8,27-31], including the steps from 5 to 9.

To operationalize this vision in practice, we aim to supplement and expand the current “The Box” service into a “Family Box,” provided that the proposed prototype features prove to be feasible and desirable after thorough testing with users. Specifically, this would involve developing a family member-facing version of the existing smartphone app (with its own login and content), in which to implement validated, personalized RPFM features. In doing so, it will be essential to prevent digital overwhelm. Patients and their loved ones recovering from and coping with cardiac events already experience cognitive fatigue and psychoemotional distress, reducing their capacity for information processing. Introducing an extensive package of digital features risks increasing this burden rather than alleviating it. Therefore, the 33 proposed features should not be implemented simultaneously but rather prioritized and bundled toward a minimal viable product-service-system. This should be developed iteratively through active co-design with patients, families, and domain experts. The features could also be delivered in a modular fashion, aligned with the patient’s and family’s stage in the care journey, ensuring that only contextually relevant support is offered at each phase. This approach to modular, journey-aligned service delivery has been explored in our previous work on family involvement in RPM-supported cardiovascular care [17], which identified heterogeneous family contexts and the need for personalized, adaptive interventions over time. Ideally, these modules would be integrated into a single digital interface to provide a seamless digital experience for both patients and their relatives, ensuring that the RPFM intervention is technically feasible, accessible, and genuinely supportive, rather than adding cognitive burden on recovering patients and their families. Beyond updates to the app, our recommendations include hospital service-level shifts, such as integrating relatives into clinical (tele-)consultation workflows and ensuring that families are included in active information provision, education, and psychosocial care throughout the entire care pathway.

While the proposed strategy originated in cardiovascular care, we find that it could be transferable to other clinical trajectories and disease areas. By following this strategy, innovators may find new opportunities for RPFM solutions that address real-life needs and achieve positive societal impact for families and health care systems.

### Strengths and Limitations

A strength of our approach lies in the integration of families’ lived experiences into a structured journey map, in alignment with Zøylner et al [55] methodology for mapping patient and relative experiences. In addition, our study expands on existing family journey mapping methodology by integrating theoretical contributions from the Family Systems Illness Model [25] and the Supportive Care Framework [26], embedded in the focus group sessions through the *Care and Well-Being Journey* template (Multimedia Appendix 2). In this way, we could distinguish between domains of family needs in a structured, theory-informed fashion. The proposed features ideated in this study offer starting points for clinicians and innovators seeking to embed family-centered RPM-supported care within cardiac care pathways. However, these are preliminary ideas, based on assumptions that require structured testing. Future research should include concept testing, pilot implementation, health economic evaluations, and studies to measure the impact on both patient-reported and family-reported outcomes and experiences. Another strength of our approach lies in its applicability to care domains other than cardiology, especially to other acute and chronic care pathways where long-term management and self-care take place at home, such as in the fields of oncology, nephrology, and obstetrics.

This study has several limitations. First, a “well-patient” and “super-user” selection bias may have affected the findings, as the sample consisted of native Dutch participants recruited from an existing RPM program (The Box), who had been enrolled for at least 2 months. Despite active and valued participation in the focus groups, participants did not react to a later chance to validate the results, which may be due to the nonbinding and possibly high-threshold nature of requesting feedback via email. The nonresponse constrains the reliability of the consensus on the ideated RPFM intervention features.

Additionally, there was a high nonparticipation rate (roughly 43%) due to reasons including illness. Consequently, the sample likely represented healthier, more engaged families, potentially introducing a risk of selection bias toward digitally literate families. This may have obscured the unmet needs of more vulnerable families who were too ill to participate or lacked the digital skills to register in the first place. Moreover, we did not collect data on socioeconomic status, educational attainment, or baseline digital literacy. Given that the proposed feature ideas are predominantly digital, this represents a significant limitation, as the proposed solution directions may prove inappropriate or inaccessible for lower-income or less digitally literate populations.

Furthermore, while this study primarily focuses on digital opportunities and on contributing to the iteration of the current version of an RPM service, the identified unmet needs might also be addressed through nondigital solutions. Examples are simplified information resources or personal, specialized extra care case managers, which may be more appropriate for less digitally literate families. Future research should intentionally include these vulnerable groups to explore alternative solution directions and ensure that RPFM interventions do not increase health disparities.

Second, there may be a recall bias that influenced the data collection, given the retrospective nature of the journey mapping. As stated before, patients were recruited at least 2 months after admission, yet they were asked to map the “Acute event” and “Admission” phases. As memory of the acute phase is often fragmented in trauma and cardiac care, we should acknowledge that reported “informational needs” during the acute phase might reflect a failure of retention due to stress rather than a failure of provision.

Third, this study merged the informational and psychological experience trajectories of the MI and peri-OP care pathways onto a single journey map. While both pathways are supported by the same RPM program, their clinical presentations and trajectories differ throughout the journey. Most notably, the peri-OP pathway involves an extended preoperative waiting period that is absent in the acute MI trajectory. Additionally, within the peri-OP pathway itself, postadmission recovery trajectories may differ considerably depending on the type of procedure, as wound healing and physical recovery following open-heart surgery differ from those following minimally invasive interventions. Merging these pathways and types of procedures onto a unified journey may have obscured pathway- and procedure-specific nuances, potentially limiting the clinical specificity and applicability of the recommendations. Future research should therefore consider testing and validating the ideated RPFM features in a pathway- and procedure-specific way, as the relevance, design, and implementation of features may differ case by case.

Fourth, the focus group sessions were small, ranging from 3 to 4 participants per session. Focus group sizes in the literature often range from 6 to 12 participants [33], and our sessions therefore fell below this recommended range. These small group sizes may have limited the interactive and synergistic dynamics characteristic of focus group methodology, potentially constraining the diversity of perspectives captured within each session.

Fifth, there is a discrepancy between the definition of “family” used, as intended by Goldfarb et al [14], and the sample included. While we advocate for an inclusive definition of family that includes friends and neighbors, our sample consisted only of familial caregivers (partners, children, and parents). The legal, privacy, and sociotechnical access needs of nonkin caregivers differ from those of immediate family members; yet our findings and features are currently based solely on immediate familial caregivers. Future work should explore whether the requirements of broader social support networks, such as neighbors or friends, align with the insights gathered from these closer familial ties.

Sixth, only one unmet need was identified in the spiritual domain in the results, which is anomalous for cardiac care research, where existential distress is often prominent. The reason for limited results in this domain may be due to patients and relatives perceiving hospitals as institutions focused on physical and psychological care and rehabilitation, rather than existential guidance or meaning. Consequently, they may be less inclined to report spiritual needs in a study focused on remote hospital care and support. Moreover, this may be due to cultural factors in the Dutch sample, where spirituality may have a relatively less prominent role in some subcultures. Future research should determine whether these needs are absent, unexpressed, or possibly addressed outside the formal care system.

Finally, the analysis and construction of the care journey may have been influenced by our individual perspectives and professional interests as medical and design professionals, particularly, our engagement with RPM and digital innovation. Our perspectives may have shaped how certain codes and themes were interpreted and prioritized. We sought to minimize the impact of individual and professional bias by engaging in in-depth consensus discussions and including reflections from transdisciplinary perspectives within the research team.

### Conclusion

By exploring the lived experiences of patients and their relatives following MI or peri-OP RPM-supported care trajectories, this study identified experience gaps in RPM-supported cardiac care, especially during preadmission and early postdischarge phases. Through our human-centered design approach, we created an overview of unmet informational, psychoemotional, social, physical, practical, and spiritual needs of families, which informed the generation of RPFM intervention feature ideas.

Our findings demonstrate that the most unmet needs regarding care and support in the RPM-supported care pathways extend beyond hospital admission and discharge. Health behaviors and recovery mostly occur at home, within the patient’s relational ecosystem with loved ones. The RPFM intervention feature ideas proposed in this study provide preliminary directions for exploration toward a transition from individually focused monitoring to inclusive, family-centered care and management.

## Supplementary material

10.2196/83055 Multimedia Appendix 1 Focus group overview.

10.2196/83055 Multimedia Appendix 2 The care and well-being journey.

10.2196/83055 Multimedia Appendix 3 Clusters of remote patient and family management interventions.

## Abbreviations

COREQ: Consolidated Criteria for Reporting Qualitative Research
CVD: cardiovascular disease
MI: myocardial infarction
peri-OP: perioperative
RPFM: remote patient and family management
RPM: remote patient management
