# Supplementary material for: Involving Families in Cardiac Care Through Remote Patient and Family Management: Focus Group and Journey Mapping Study
Source: JMIR Cardio. 2026 Jul 28;10:e83055. doi: 10.2196/83055 (PMC13412015; doi:10.2196/83055)
Supplement: Multimedia Appendix 1 [file cardio-v10-e83055-s001.docx]

| **Focus group** | **CVD care path** | **#** | **Type** | **Relation to patient** | **Sex** | **Age Group** |
| --- | --- | --- | --- | --- | --- | --- |
| F1.A | Peri-OP | 1 | Patient | - | F | 70-80 |
|  |  | 2 | Patient | - | M | 65-75 |
|  |  | 3 | Patient | - | F | 50-60 |
|  |  | 4 | Patient | - | F | 30-40 |
| F1.B | Peri-OP | 5 | Relative | Father | M | 75-85 |
|  |  | 6 | Relative | Child | F | 45-55 |
|  |  | 7 | Relative | Partner | F | 55-65 |
|  |  | 8 | Relative | Partner | M | 30-40 |
| F2.A | MI | 9 | Patient | - | F | 50-60 |
|  |  | 10 | Patient | - | F | 70-80 |
|  |  | 11 | Patient | - | F | 55-65 |
| F2.B | MI | 12 | Relative | Father | M | 75-85 |
|  |  | 13 | Relative | Partner | M | 70-80 |
|  |  | 14 | Relative | Child | M | 20-30 |
| F3 | Peri-OP | 15 | Patient | - | M | 70-80 |
|  |  | 16 | Patient | - | M | 60-70 |
|  |  | 17 | Relative | Partner | F | 55-65 |
| F4.A | MI | 18 | Patient | - | F | 60-70 |
|  |  | 19 | Patient | - | M | 55-65 |
|  |  | 20 | Patient | - | F | 35-45 |
|  |  | 21 | Patient | - | M | 55-65 |
| F4.B | MI | 22 | Relative | Partner | F | 55-65 |
|  |  | 23 | Relative | Partner | F | 55-65 |
|  |  | 24 | Relative | Partner | M | 40-50 |

## Multimedia Appendix 1 – Focus Group Overview
