# Supplementary material for: Involving Families in Cardiac Care Through Remote Patient and Family Management: Focus Group and Journey Mapping Study
Source: JMIR Cardio. 2026 Jul 28;10:e83055. doi: 10.2196/83055 (PMC13412015; doi:10.2196/83055)
Supplement: Multimedia Appendix 2 [file cardio-v10-e83055-s002.docx]

## Multimedia Appendix 2 – The Care and Well-Being Journey

We designed and employed a journey mapping template called *The Care and Well-Being Journey* to guide the discussions, see Figure 5 below.


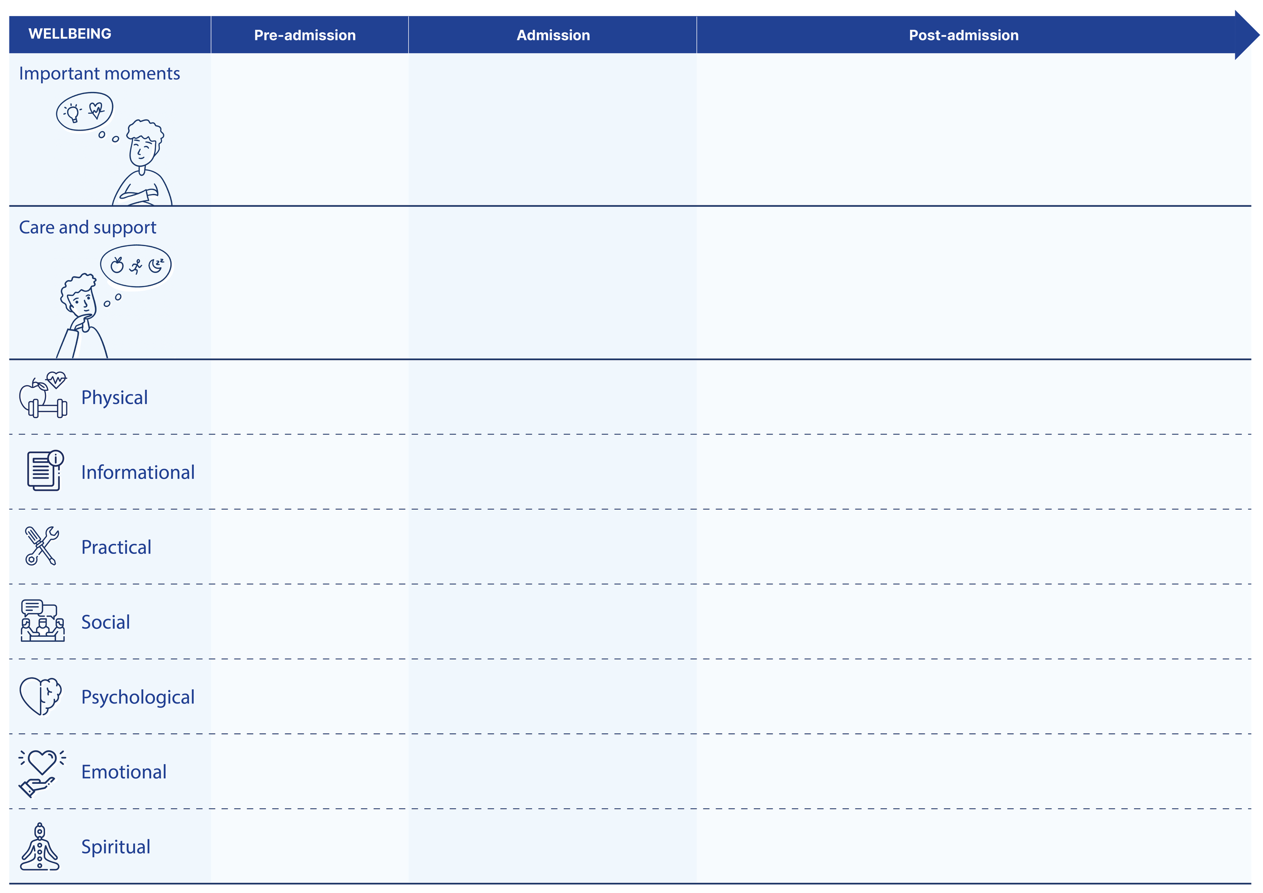


*Figure 5. The Care and Well-Being Journey*

The semi-structured focus group interview guide included the following main questions:

- What key moments (positive and negative) in the cardiac disease journey have stayed with you, and how did you experience them?
- In what ways has there been support and care for your well-being both as individuals and as families from the care system?
- Do you feel responsibility/involvement towards each other regarding health and well-being? And to what extent are/were you able to help each other?
- What improvements could help the whole family navigate the journey, and what form could they take (e.g. coaching, tools, extra help)?
